# The acute effect of training on hip adduction strength within a football microcycle

**DOI:** 10.5114/biolsport.2026.159562

**Published:** 2026-04-20

**Authors:** Maziar J. Hamad, Pedro E. Alcaraz, Kristian Thorborg, Steve Barrett, Konstantinos Spyrou

**Affiliations:** 1 UCAM Research Center for High Performance Sport, Catholic University of Murcia (UCAM), Murcia, Spain; 2 Faculty of Sport Sciences, Catholic University of Murcia (UCAM), Murcia, Spain; 3 Strength and Conditioning Society (SCS), Murcia, Spain; 4 Department of Sports, Department of Orthopedic Surgery, Orthopedic Research Center-Copenhagen (SORC-C), Amager-Hvidovre Hospital, Copenhagen University Hospital, Copenhagen, Denmark; 5 Playermaker, London, United Kingdom; 6 Global Institute of Sport, Manchester, United Kingdom

**Keywords:** Injury, Isometric contraction, Groin

## Abstract

This exploratory study aimed to examine the changes to hip adduction strength within the football microcycle. The microcycle included the following training sessions: match day minus 4 (MD-4), MD-2 and MD-1. Twelve male youth football players were recruited (age: 17.11 ± 0.52 years; body mass: 72.63 ± 5.53 kg; height: 1.78 ± 0.05 m) and had their maximal isometric hip adduction strength (ADD_iso_) measured before and immediately after each training session. Players wore foot-mounted inertial measurement units (Playermaker) during each training session to quantify locomotor activities. A linear mixed model and one sample t test were used to assess ADD_iso_ and percent changes, respectively. Statistical significance was set at ≤ 0.05. The group arrived at MD-1 with the lowest strength of the week, but finished the training with recovered ADD_iso_, similar to all other pre-match time points (MD-4_pre_, 3.04 Nmkg^–1^; MD-2_pre_, 3.09 Nmkg^–1^; MD-1_pre_, 3.04 Nmkg^–1^, p > 0.05). The group-level post-training percent changes to ADD_iso_ were: MD-4, –4.00 ± 7.29%, p = 0.083; MD-2, –3.76 ± 5.04%, p = 0.043; and MD-1, 7.43 ± 4.92%, p < 0.001. In summary, high-intensity training sessions with high-speed running and change of direction actions were associated with reductions of hipadduction strength, while sessions with less high-intensity locomotor demands while emphasising shooting actions were associated with a potentiating effect on ADD_iso_. This is important considering the relationship between hip adduction strength and groin injury and pain.

## INTRODUCTION

In football, the structure of a microcycle is important to optimally prepare a team for an upcoming match [1, 2]. Within the competitive season, many teams implement similar microcycle structures (i.e. tactical periodization), whereby the type and sequence of training sessions are manipulated to adequately emphasize recovery early in the microcycle (match-week), an increased training stimulus midweek, followed by tapering or priming sessions in the final days before a match [1–3]. As this weekly periodization strategy has been shown to impose notable differences in internal and external loads between training days [2, 4], this may signify that performance and injury risk profiles of players vary within the microcycle (i.e. MD-4 vs MD-1). Therefore, to quantify the training response and assess readiness, practitioners may at times implement monitoring strategies to assess a player’s neuromuscular performance [5].

The monitoring of neuromuscular function within football is commonly used to understand a player’s physical performance, readiness to train, and their injury risk profile [5, 6]. As one such example, the use of hip adduction strength testing can be an important metric for assessing hip and groin performance, as previous research has reported significant relationships between hip adductor strength and performance in kicking [7], 10 and 20 m sprinting, jumping, and 180° changes of direction [8]. The use of hip adduction strength testing has also been proposed as a secondary prevention tool [9], as it can help identify players with reduced groin function, pain [10], and those with an increased risk of groin injury [11, 12]. The implementation of hip adductor testing at different time points and settings, such as at pre-season [12], during tournament settings [13, 14], and at weekly time points throughout a competitive season [10, 15], has improved the understanding of temporal changes during the season and under congested-fixture conditions.

While periodic hip adduction strength testing within screening and monitoring practices can inform coaches about injury risk [9, 10], testing at longer time points may miss the acute and residual changes which may have occurred within the football microcycle. While research is increasing surrounding post-match hip adduction strength responses [16–18], data are more scarce regarding the acute response after football training [19]. For example, Buchheit and colleagues observed that training sessions that emphasised concentrated playing areas and a greater number of changes of direction resulted in the greatest reductions in hip adduction strength. Similarly, when videos of acute hip adductor injuries were systematically analysed in football [20, 21], the most common injury mechanism was a rapid and intense eccentric action of the hip adductors that was most commonly seen in kicking, reaching, and change of direction actions. Therefore, understanding how acute fatigue and locomotor and technical demands influence the hip adductors within a football microcycle would help practitioners assess how hip and groin function respond and recover to different training prescriptions. This understanding would also provide objective data to inform player monitoring practices and thus help to optimize microcycle planning to prepare players for upcoming competitions.

Therefore, the objectives of this study were to (i) understand the time course changes in hip adduction strength within a microcycle in youth football players and (ii) quantify the immediate pre- to posttraining changes.

## MATERIALS AND METHODS

### Study design

The following article follows the STROBE Statement and its accompanying checklist to guide the reporting of an observational study [22]. The study was a descriptive study with a pre-post design aiming to investigate the post-training changes to hip adduction strength in youth football players within the microcycle during the 2024/2025 competitive season. Maximal isometric hip adduction strength (ADD_iso_), and subjective internal and external training demands were measured during the week. To familiarize the participants with the study and testing procedures, two familiarization sessions were held at the same time and day, one week apart. For the study, participants were tested 15 minutes before the start of training (Pre), and immediately after the termination of the session (Post). The training sessions took place on Tuesdays, Thursdays, and Fridays, corresponding to match days (MD) MD-4, MD-2 and MD-1, respectively (Table 1). Because of the need to test players immediately after the session to capture acute neuromuscular fatigue, only six players were measured in the first week, followed by the remaining six in the subsequent week. The training plans for each session were identical between the two weeks of testing and are provided in Table 1.

**FIG. 1:**
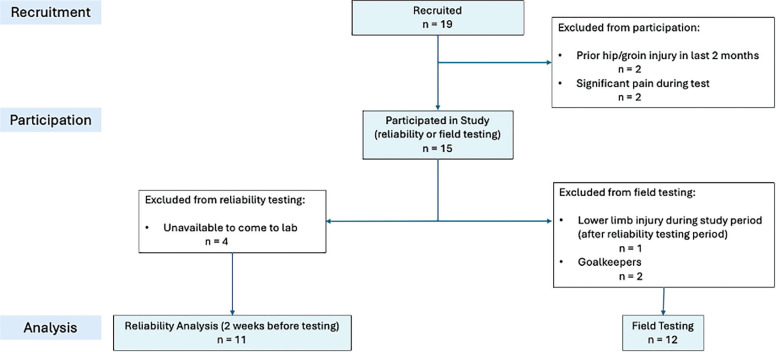
Subject screening process.

**FIG. 2:**
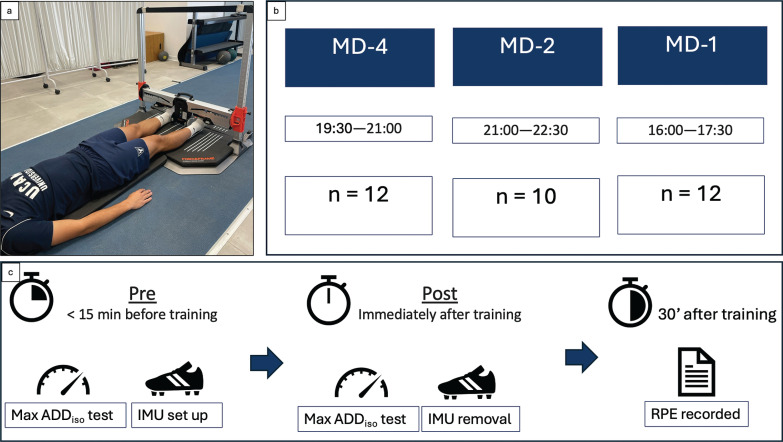
Study design: a, Measurement of ADDiso; b, Matchday time points and sample sizes; c, Pre-post testing procedure

**TABLE 1:** Detailed training programs during the microcycle^*^.

MD-4	MD-2	MD-1
15 m run with zig-zag change of directions	10–15 m unresisted and resisted accelerations	Low-intensity tactical set-piece walk-throughs

Possession 5 v 3 (15 m × 15 m) followed by 20 m shuttle when the larger team lost possession	Unresisted 35 m sprints Rondos 4 v 1 (4 × 4 m)	Low-intensity phases of play walk-throughs (ex. building out from the back)

Possession 8 v 5 (39 m × 28 m)	Tactical organization drills (52 m × 64 m)	Possession 5 v 5 (+5 neutrals along perimeter and 1 inside) (26 m × 40 m)

Game with attack, and repeated counterattacks (including goalkeepers) ex. 1 v 0, 1 v 1, 2 v 1, 2 v 2. (37 m × 39 m)	Possession 6 v 6 (+2 neutrals on perimeter and 1 inside) (47 m × 40 m)	Uncontested shooting drills around the box

Game 4 v 4 (+4 neutrals on perimeter) (24 m × 29 m)

* The training program was identical for the two weeks of data collection.

### Participants

To qualify for inclusion in this study, subjects had to be healthy, without a previous hip and groin injury within the past 2 months and be capable of performing the maximal strength tests without significant pain. Nineteen professional outfield players were recruited for participation. Four players were excluded as they had either significant pain during tests (n = 2) or had a previous groin injury in the past 2 months (n = 2). One player who participated in the familiarization period suffered a lower limb injury a week after and was therefore excluded from the training data. Two goalkeepers were also excluded from the training data collection. Therefore, 12 healthy youth football players (age: 17.11 ± 0.52 years; body mass: 72.63 ± 5.53 kg; height: 1.78 ± 0.05 m) participated in the study. Subjects were involved in approximately 7.5 hours of structured training per week and had at least 4 years of competitive football experience and at least 2 years of structured resistance training experience. Two players were unavailable to come to training on two occasions due to being called up to the older team; therefore, sample sizes at MD-4, MD-2, and MD-1 were n = 12, n = 10, and n = 12, respectively. All subjects were informed about the procedures, details, and risks of the study, and all provided their written consent. Legal guardians signed the informed consent for any participant who was a minor. The study was completed according to the 2013 Declaration of Helsinki, and ethical approval was provided by the local Ethics Committee (registration number: CE042410).

#### Isometric hip adduction strength (ADD_iso_)

To assess maximum isometric hip adduction strength (ADD_iso_), measurements were taken before and after training in a supine position. Before each maximal test, players performed a warm-up repetition progressively going from 50% to 80% of perceived maximal intensity for 5 s. Following the warm-up, participants performed 3 maximal, 5-s hip adduction efforts, interspersed with 30 s of rest. Subjects were instructed to push inwards on the sensors as hard as possible, and verbal encouragement was provided during each repetition. ADD_iso_ was measured using a fixed dynamometer (ForceFrame, VALD Performance, Brisbane, Australia) by the same tester each time. For each repetition, the left and right peak forces were averaged, and the best trial was kept and converted to relative torque (Nmkg^–1^) using each participant’s limb length (m) and body mass (kg). Limb length was measured from the anterior superior iliac spine to the medial malleolus (point of application of force). The inter-day reliability of the fixed dynamometer was tested in-house with 11 players, under the same conditions (day and time), one week apart. The intraclass correlation corresponding to ICC_(3,1)_ and its 95% CI were: 0.88 (0.63–0.97). Mean (± SD) within-subject coefficient of variation was 4.93 ± 3.26%. The standard error of measurement (SEM) was calculated as $\text{SD}\times \sqrt{1-\text{ICC}}$ [23], and used to determine the SEM%

$$\frac{\text{SEM}}{{\text{Mean}}_{\text{pooled}}}$$

The SEM% was then used to determine the minimal detectable change (%) at an individual (MDC_ind_) ($\text{SEM}\%\times 1.96\times \sqrt{2}$) and group level (MDC_group_) $\frac{\text{SEM\%}\times 1.96\times \sqrt{2}}{\sqrt{\text{n}}}$ [23, 24].

The MDC_ind_ was 13.31% and the MDC_group_ was 4.01%.

#### Internal and external training demands

To assess subjective internal demands, rating of perceived exertion was collected from each player, 30 minutes after each training session [25] and was later multiplied by the session duration to give a sessional rating of perceived exertion (sRPE). To assess the different mechanisms that could impact the hip and groin region, locomotor and technical demands during each training session were measured using foot-mounted inertial measurement units (IMU) (Playermaker, London, UK). The IMUs contained a 3-axis gyroscope and 3-axis accelerometer to quantify the angular velocity and acceleration, respectively, of each foot. Each IMU is within a silicone sleeve that is worn on each football cleat during the training session. The Playermaker has shown good to excellent between-unit reliability [26] and good validity for measuring locomotor characteristics [27]. Additionally, each player wore the same IMU throughout the study to minimize inter-unit reliability issues. The locomotor variables were: distance covered (m), high-intensity distances > 4 ms^–1^ (HID) (m), sprint distances > 5.5 ms^–1^ (SpD) (m), total horizontal acceleration distance (m), total horizontal deceleration distance (m), and intense speed change actions (e.g., accelerations and decelerations) > 2.6 ms^–2^ (#). Technical actions were also quantified, and the variables measured were the total number of releases (passes and shots) (#) and the release index (average release velocity × total number of releases)/100. The release index has been previously used as a metric to collectively reflect the volume and intensity of all the kicking actions [28].

### Statistical analyses

All data were first confirmed for normality using the Shapiro-Wilk test. Descriptive data are presented as mean ± SD. Statistical significance was set at ≤ 0.05, and the analyses were conducted in JASP (Version 0.18.3) [29].

To assess time-course changes in relative torque, a linear mixed model was used. The model terms were tested with the Satterthwaite test method, with each time point (i.e. MD-4 pre, MD-4 post, MD-2 pre, etc.) used as a fixed effect and each participant as a random effect grouping factor. If any statistically significant main or interaction effects were found, a post hoc analysis was performed with a Bonferroni adjustment. A one sample t-test was conducted on the pre- to post-training percent change in strength for each MD time point to determine whether any changes differed from the null hypothesis (= 0). Effect sizes and their 95% confidence intervals [95% CI], are reported as Hedges’ g_av_ following the guidelines of Lakens [30]. Effect sizes were interpreted as follows: < 0.2, trivial; 0.2–0.6, small; 0.6–1.2, moderate; 1.2–2.0, large; > 2.0, very large [31].

A sensitivity analysis was performed in G*Power (version 3.1.9.6, University of Dusseldorf, Dusseldorf, Germany) to compute the minimum effect size needed for a two-tailed t test with a type I error rate of 0.05 and a power of 0.80, given a minimum sample size of 10. The required effect size for the pre-post change in strength was 1.00.

## RESULTS

To confirm whether the training was identical between weeks, an independent sample t-test was conducted to assess the training demands between the two consecutive training weeks. There were no significant differences between the two weeks for any of the locomotor actions (*p* > 0.05). A summary of the internal and external demands for each training MD is presented in Table 2.

**TABLE 2:** Group subjective-internal demands and locomotor and technical actions.

Variables	MD-4	MD-2	MD-1
sRPE	485 ± 51	562 ± 148	390 ± 38
Releases (#)	95 ± 26	50 ± 17	59 ± 10
Release index	90 ± 22	56 ± 18	66 ± 13
Distance covered (m)	5116 ± 1017	5576 ± 607	3559 ± 335
HID covered (m)	574 ± 223	790 ± 311	161 ± 70
SpD covered (m)	111 ± 82	263 ± 94	15 ± 10
Intense speed changes (#)	66 ± 17	60 ± 15	32 ± 10

Summary data is presented as mean ± SD, HID, high intensity distances (> 4 ms^–1^); Intense speed changes are defined as accel/decel actions > 2.6 ms^–2^; SpD, sprint distances (> 5.5 ms^–1^).

**FIG. 3:**
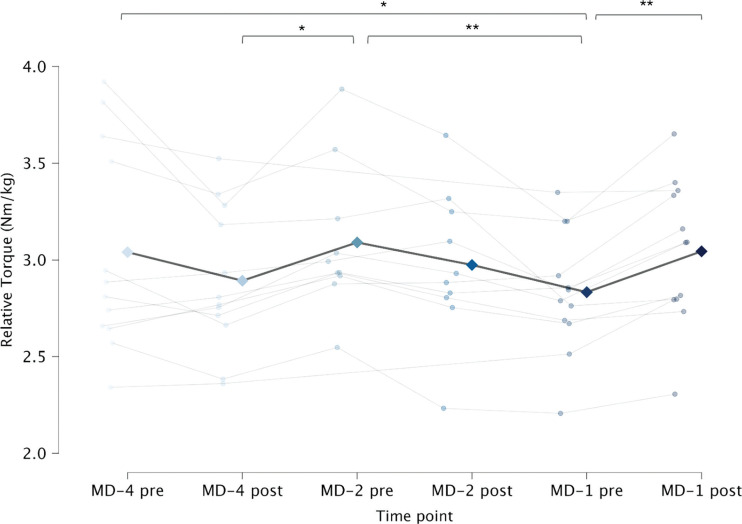
Relative torque (N·m·kg^–1^) of maximal isometric hip-adduction at different time points. Points represent individual data; diamonds represent group means. * p < 0.05; ** p < 0.01

For the assessment of the effect of the MD (i.e. MD-4 pre, MD-4 post, MD-2 pre, etc.) on strength, the result of the linear mixed model showed a significant effect of time point on relative torque, F_(5, 51.01)_ = 3.523, *p* = 0.008. Post-hoc analysis showed that MD-1_pre_ strength was significantly lower than MD-4_pre_ (mean diff: –0.21 Nmkg^–1^, ES = –0.54 [1.12, 0.06], *p* = 0.028) and MD-2_pre_ (mean diff: –0.24 Nmkg^–1^, ES = –0.86 [–1.55, –0.13], *p* = 0.004); and MD-1_post_ was significantly higher than MD-1_pre_ (mean diff: 0.21 Nmkg^–1^, ES = 1.39 [0.60, 2.16], *p* = 0.006). Secondly, MD-2_pre_ was significantly higher than MD-4_post_ (mean diff: 0.21 Nmkg^–1^, ES = 0.30 [–0.32, 0.90], *p* = 0.047). The relative torques and group means for each timepoint are presented in Figure 3.

**FIG. 4:**
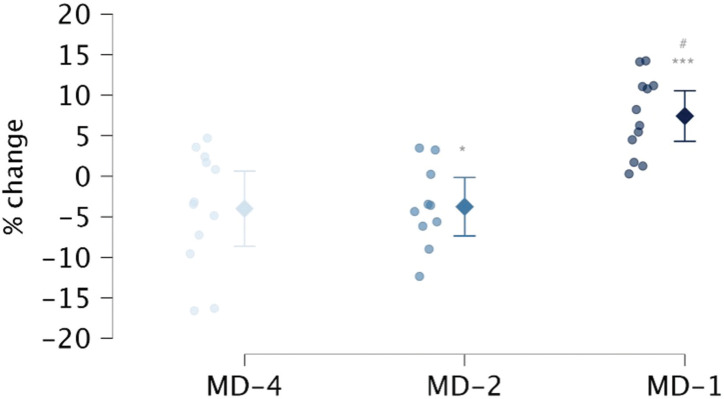
Pre-post percent change in ADDiso. Points represent individual-player percent changes. Diamonds represent group means and their 95% confidence intervals. Significant percent change, * p < 0.05, *** p < 0.001. MD-1 changes significantly different from all other time points, # p < 0.001

The result of the one-sample t-test showed that there was a significant reduction in strength at MD-2, (–3.76 ± 5.04%, ES = –0.73 [–1.39, –0.03], t(9) = –2.360, *p* = 0.043), and a significant increase in strength at MD-1 (7.43 ± 4.92%, ES = 1.39 [0.60, 2.16] t(11) = 5.231, *p* < 0.001). The changes in strength at MD-4 (–4.00 ± 7.29%, ES = –0.55 [–1.13, 0.05]) were not statistically significant, (t(11) = 1.904, *p* = 0.083). There was a significant effect of the time point on percent change in strength, F_(2, 21.14)_ = 14.641, *p* < 0.001; the post-hoc analysis revealed that the percent change in MD-1 was significantly greater from MD-4 and MD-2 (*p* < 0.001). The individual and group percent changes are displayed in Figure 4.

## DISCUSSION

The aim of the present study was to determine the time-course changes to hip adduction strength that occur within the microcycle of youth football players and to quantify the pre-post percent change from training sessions. The main findings of the study were that: (i) in this sample of youth footballers, MD-1_pre_ displayed the lowest average ADD_iso_ of all the pre-training time points, but by the posttraining time point it had recovered; (ii) there were contrasting strength changes depending on the training day, with average group responses ranging from 4% reductions to 7% improvements. While there was a non-significant reduction in strength at MD-4 (–4.00%) and a significant reduction at MD-2 (–3.76%), only the 7.43% improvement on MD-1 was above the minimum effect size from the sensitivity analysis.

Regarding the recovery of ADD_iso_, it seems that at a group level, pre-training strength, measured each training day, was not fully recovered at MD-1_pre_. This may in part be because less recovery time was available between the previous day’s session (< 18 hours) in comparison to the 48 hours between the other time points (e.g. MD-4_post_ and MD-2_pre_). When considering that 48 hours later, MD-4_pre_, strength was not significantly different from MD-2_pre_, and in light of similar findings in the literature [16, 17], one can be reasonably confident that by 48 hours after training, ADD_iso_ strength can be recovered in this population. In fact, in a similar study we observed that ADD_iso_ had consistently recovered by 24 hours after a match in youth football players [18]. While different recovery profiles have been observed for isometric, concentric and eccentric strength [32], more research is needed to conclusively state whether 24–48 hours is also sufficient for the full recovery of hip adduction strength or whether these findings are unique to the task. While in some cases, 24 hours may be sufficient for the recovery of isometric strength, it may also depend on the preceding activity [32, 33] and its dosage [34]. This is important when considering the common injury situations when acute adductor injuries occur [20, 21]. When video footage of acute hip adductor injuries in football players were systematically analysed [20, 21], the common fundamental mechanism was a rapid action (i.e. kicking, reaching, change of direction) associated with high activation of the muscles while rapidly lengthening [35]. Therefore, it would be of interest to researchers and practitioners for future research to also investigate the recovery profile of eccentric hip adductor strength after football training and matches.

Overall, the reductions seen in this study were slightly less than some magnitudes seen after football matches (12–22%) [16–18]. In a similar study by Buchheit et al. [19], the authors observed reductions in strength between 7–12% after tactically periodized training sessions in elite football players. Similar to our findings, they also found that the greatest reductions in groin strength occurred on the first ‘acquisition’ or loading day of the microcycle, which under the tactical periodization model is defined as the ‘strength’ session [3]. Regarding individual-level changes, on three occasions pre-post changes were less than the commonly referenced individual minimal detectable changes (MDC_ind_) of 13–15% [13, 36] (Figure 3). When considering group-level MDC of approximately 4–5% [24], the pre-post changes seen in this study were greater than the MDC at a group level only on MD-1. Furthermore, when youth football players were screened for hip adduction strength and pain during a season, Delang et al. [10] observed that in the group that developed groin pain, the average percent change in strength from baseline to the week before pain onset was –9.7%, and from baseline to pain onset was –16.7%. It may be important to note that while only some individuals experience meaningful reductions after a singular training session, repeated loading of certain session types (similar to MD-4 and MD-2 of this study) without adequate recovery may lead to cumulative fatigue and reductions that approach clinical significance [10]. This is further supported by findings that have shown clinically significant hip adduction strength reductions in youth football players during congested periods [13].

In this study the within-day pre-post changes in ADD_iso_ had contrasting responses depending on which training session was performed. Two of the three training sessions (MD-4 and MD-2) resulted in decreases in strength on average, while one resulted in an increase in strength (MD-1). This finding—that the groin region was stressed differently depending on the training session—is consistent with how many teams organize their in-season microcycle [2, 4], such that the session emphasis can vary between recovery, loading, or priming. It is then of interest to understand which types of sessions cause the greatest reduction in groin strength and whether a dose-response relationship could exist between the demands (type, intensity, and quantity, and types of locomotor and technical actions performed) and the hip adduction strength response. The neuromuscular response to matches in football was previously investigated in a meta-analysis by Hader et al. [37], and it was found that match-related running distance above 5.5 ms^−1^ (classified as SpD in our study) was strongly negatively correlated with post-24 hour jumping performance. In our study, considering the contrasting strength responses seen on MD-2 (–4%) and MD-1 (+7%) and the large differences in SpD for MD-2 (263 m) and MD-1 (15 m), it is possible that differences in session demands and locomotor actions may explain this finding. Since hip adductor injuries have been shown to occur during certain defined actions [20, 21], we suggest that further research should explore whether a relationship exists between reductions in ADD_iso_ and common locomotor and technical actions, such as high-speed running, change of directions, and kicking.

An interesting finding from this study is that even though MD-1_pre_ presented the lowest strength of the week (2.83 Nmkg^–1^), by the end of the session (MD-1_post_), strength significantly increased by 7%, to 3.04 Nmkg^–1^ (*p* < 0.001), which was not significantly different to MD-4_pre_ (3.04 Nmkg^–1^) or MD-2_pre_ (3.09 Nmkg^–1^) (*p* > 0.05). This finding is interesting, since it may show that despite not arriving fully recovered, some training sessions and actions may provide an acute priming or compensatory effect to ADD_iso_ [18, 38, 39]. In fact, acute increases in hip adduction strength were also previously found at 24 hours [18] and 48 hours [38] after a match in football and rugby, respectively. The acute 7% post-activity improvement of hip adduction strength observed in the present study is also in line with previous research by Jensen et al. [39], who found a near 8% increase in isometric hip adduction strength 24 hours after a repetitive kicking drill in youth football players. In addition to having an emphasis on uncontested shooting drills, the MD-1 training session in our study also had the lowest sRPE of all the training sessions. This finding may support the prescription of low-intensity training sessions that involve kicking/shooting actions as a pre-match or MD-1 session to increase acute performance of the hip adductors and possibly reduce injury risk [11, 12].

### Limitations

The current study had some limitations that need to be considered when interpreting the findings. Firstly, the specific sample of youth football players limits the ability to generalize the findings to other populations. Secondly, the small sample size in this study meant that detecting small to moderate changes was limited and that, according to our sensitivity analysis, the current study was only sufficiently powered to detect large effects. Furthermore, due to the small sample size in the current study, the precision of the effect estimates is low, as reflected in the wide confidence intervals. Additionally, the specific 3-day training organization of the team’s microcycle means that these findings may not be generalizable to other in-season training models employed by teams that include different training frequencies and programmes. Finally, another limitation of this study was that only one contraction mode (isometric) was tested, and therefore the findings are not translatable to the recovery of eccentric hip adduction strength.

## CONCLUSIONS

The main conclusions of the current observational study were that hip adduction strength remained stable across the microcycle consisting of three training days in youth football players. The pre-post percent changes to hip adduction strength seemed to depend on the training session, as the ‘loading’ days earlier in the week (MD-4, MD-2) were associated with reductions in strength, while the less intense MD-1 session was associated with an increase of hip adduction strength.

The programming of training sessions in youth football may influence the response of hip adduction strength in season. Different pre-post changes may be seen in strength depending on the training day or emphasis of the session. Higher intensity training sessions earlier in the week that emphasize greater high-speed running and change of direction actions may result in reductions of hip adduction strength. In contrast, sessions with fewer high-intensity actions while still emphasising shooting actions may provide a potentiating effect and increase hip adduction strength. This is important considering that hip adductor strength is a modifiable risk factor for groin injuries and the relationship between hip adduction strength and groin pain.

## Data Availability

The data supporting the findings of this study are available upon reasonable request from the corresponding author [MJH].
